# Effect of NPK Fertilizer on Chemical Composition of Pumpkin (*Cucurbita pepo* Linn.) Seeds

**DOI:** 10.1100/2012/808196

**Published:** 2012-05-02

**Authors:** F. M. Oloyede, I. O. Obisesan, G. O. Agbaje, E. M. Obuotor

**Affiliations:** ^1^Department of Crop Production and Protection, Obafemi Awolowo University, Ile-Ife, Nigeria; ^2^Department of Biochemistry, Obafemi Awolowo University, 220005 Ile-Ife, Nigeria

## Abstract

An investigation of the proximate composition and antioxidant profile of pumpkin seeds obtained from different levels of NPK 15 : 15 : 15 compound fertilizer application at the Obafemi Awolowo University, Ile-Ife, Nigeria was carried out. Pumpkin seeds were grown in 2010 for two cropping seasons (May to August and August to November), and the following fertilizer rates were applied: 0, 50, 100, 150, 200, and 250 kg/ha. Standard analytical methods were used to determine protein, crude fibre, ash, fat, carbohydrate, antioxidant activities, phenol, flavonoid, proanthocyanidin, and anthocyanin. The highest concentrations of the proximate and antioxidants analysed were found from the seeds of control and those treated with lower NPK rates. The mean protein, ash, crude fibre, and carbohydrate values of pumpkin seeds at zero to 100 kg NPK/ha were 27%, 1.56%, 0.56%, and 11.7% respectively. At these same levels of fertilizer, pumpkin seed oil yield was 59%. Antioxidant activities ranged from 89.9 to 90.4% while total phenol was 47 mg/100 g. Except for carbohydrate, the % concentration of nutrients and antioxidants in pumpkin seeds was significantly (*P* = 0.05) depressed with fertilizer rates above 100 g/ha.

## 1. Introduction

Pumpkin seed vegetable is valued in many countries such as Japan, Czech Republic, Hungary, Germany, Austria, Romania, Italy, and West and South Ukraine. The seeds are a good raw material for the production of oil used in food preparation and in medicine [1, 2]. According to Jariene [3], the seeds contain about 50% fats, approximately 30% protein, sugar, B vitamins, ascorbic acid, Phytosteroles, Phytin, lecithin, oxycerotine, tyrosine, salicylic acid, and resins. The seed oil is also rich in glycerides of linoleic, oleinic, palmitin, and stearine acids. Omega-3 fatty acid was found to be present in pumpkin seeds; it helps to prevent artheriosclerosis, high blood pressure, and heart diseases; it also stimulates metabolism of accumulated fats. Oil-cake fats from pumpkin seeds contain large amounts of (almost 60%) of omega-3 acids twice that of cod liver oil [3–6]. Pumpkin seed powder is used in China and the United States as an ingredient of salad dressings and in baked products. The seed oil is used as salad oil in Europe, and in India for cooking and lighting. The seed is used medicinally in the prevention of kidney stones. Seeds are eaten as an anthelmintic. In Mauritius an infusion of the seeds is used internally to treat hypertension and prostate complaints and externally to treat erysipelas [7].

Seeds form a major part of the diet of Nigerians; They are consumed as a meal as well as ingredients of local soups. In southwestern Nigeria, pumpkin seeds are used locally as an alternative to “egusi” melon (*Citrullus vulgaris* Schrad) seed. Melon seeds are milled and used to prepare the popular “egusi” soup where they act as food thickeners. Pumpkin seeds are used alone or in combination with leafy vegetable. 

Information is scanty on the nutrient and antioxidant composition of pumpkin seeds in Nigeria. Due to reduction or loss of soil fertility in most Nigerian soils, chemical fertilizers are used to boost crop yield; this consequently has bearing on the chemical composition of the crops grown on such lands. This study thus aimed at evaluating the influence of NPK fertilizer on the proximate composition and antioxidant profile of pumpkin seeds.

## 2. Materials and Methods

### 2.1. Field Study

Pumpkin fruits were harvested after 15 weeks at the Teaching and Research Farm, Obafemi Awolowo Univerity, Ile-Ife, Nigeria for 2 seasons in 2010. The experiment was a randomized complete block design consisted of 6 rates of NPK 15 : 15 : 15 fertilizer at 0, 50, 100, 150, 200, and 250 kg/ha. There were 6 replicates of 10 m × 12 m plot size. At maturity, 5 fruits each at random from all the plots were chosen and their seeds extracted, rinsed, and dried at 50°C. Six composite samples from the 6 replicates were milled and stored in the refrigerator. 

### 2.2. Laboratory Analyses

For the antioxidant assays, about 5 g each of the composite samples were extracted by cold extraction, that is, extraction not involving heat, for 24 hours using 80% methanol. The crude extract was obtained by evaporation of the methanol soluble extract to dryness. The hydrogen donating or radical scavenging of the extract was determined using the stable radical DPPH (2,2-diphenyl-2-picrylhydrazyl hydrate) according to the method described by Brand-Williams et al. [8]. DPPH reacts with an antioxidant compound which can donate hydrogen, and it is reduced. The change in colour from deep violet to light yellow was measured spectrophotometrically at 517 nm. Total phenol content was determined by the method of Singleton and Rossi [9] using the Folin-Ciocalteau reagent in alkaline medium. Total flavonoid content was determined using AlCl_3_ method as described by Lamaison and Carnet [10]. The proanthocyanidin content was determined using a modified method of Porter et al. [11] using the AlCl/Butan—1-0l assay method. The total anthocyanin content of the test samples was determined using the pH differential method of Fuleki and Francis [12]. Crude protein, Carbohydrate, Ash, Crude fibre, Ether extract (fat), and Moisture contents were determined using the routine chemical analytical methods of Association of Official Agricultural Chemists (AOAC) [13]. All data were subjected to combined analysis of variance SAS [14]. Means squares, significantly different, were separated using Duncan Multiple Range Test (DMRT) at 5% level of probability.

## 3. Results

**Figure 1:**
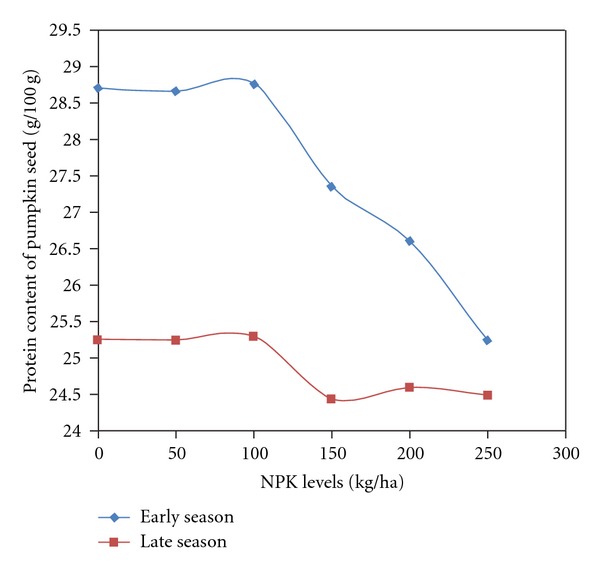
Protein content of pumpkin seeds as affected by season by NPK fertilizer.

**Figure 2:**
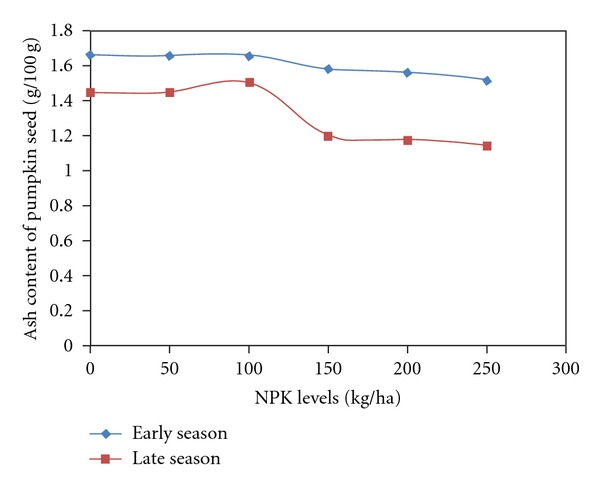
Ash contents of pumpkin seeds as affected by season by NPK fertilizer.

**Figure 3:**
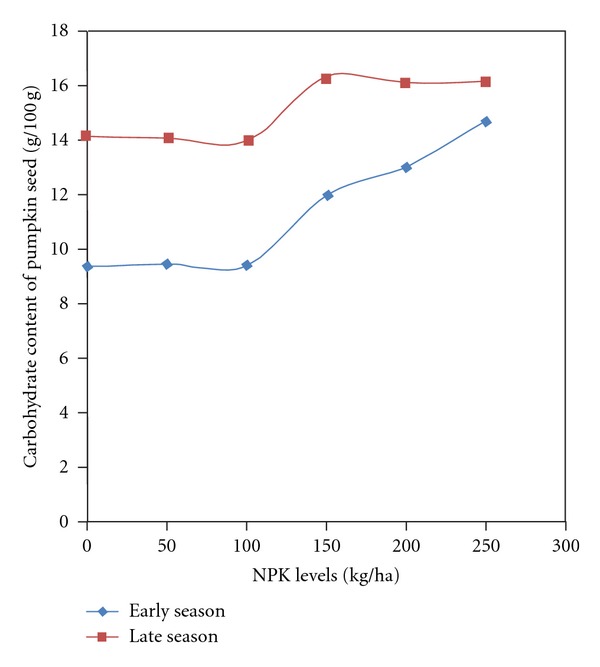
Carbohydrates content of pumpkin seeds as affected by season by NPK fertilizer interaction.

The protein, fat, ash, crude fibre, and carbohydrate in pumpkin seeds were significantly influenced by season and fertilizer. Interaction between season and fertilizer was significant on protein, ash, and carbohydrate contents (Table 1). Seasonal influence showed higher nutrient values during the early season than in the season except for carbohydrate. Variation between the nutrient values ranges from 1.4 to 25%. Protein was 10% higher in early season while carbohydrate in late season was higher by 25% than in the early season (Table 2). Fertilizer influence showed that proximate values of protein, fat, ash, and crude fibre in pumpkin seeds were similar at 0, 50 and 100 kg NPK/ha. The nutrient values reduced significantly when compared to the control from the application of 150 kg/ha of NPK and this continued to the highest fertilizer rate (250 kg NPK/ha) with the exception of carbohydrate content whose values increased with increased fertilizer rates. Carbohydrate concentration in pumpkin seeds was highest with the application of 250 kg/ha (Table 3). The influence of season and fertilizer interaction on protein and ash contents showed that the nutrient concentration was higher across fertilizer rates in the early season than the late season. The values were significantly lower than in the control when fertilizer application was above 100 kg/ha both in the early and in the late seasons (Figures 1 and 2). On the contrary, the carbohydrate content of pumpkin seeds was higher in late season than that in the early season (Figure 3).

**Figure 4:**
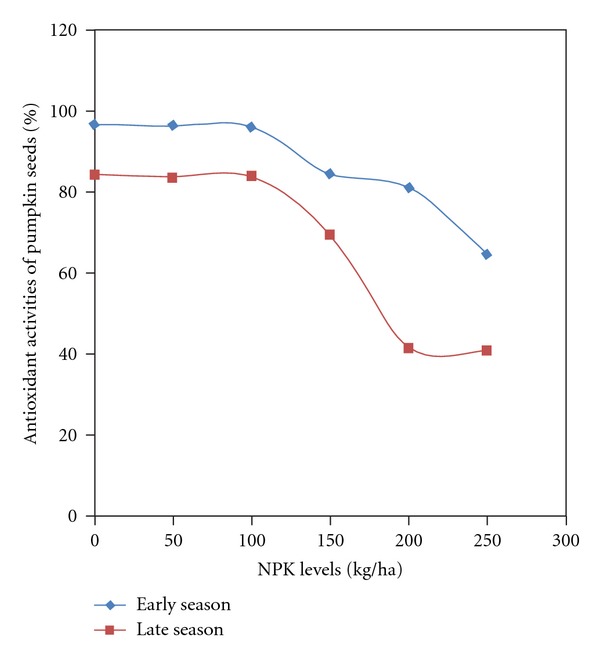
Antioxidant activities of pumpkin seeds as affected by season by NPK fertilizer interaction.

**Figure 5:**
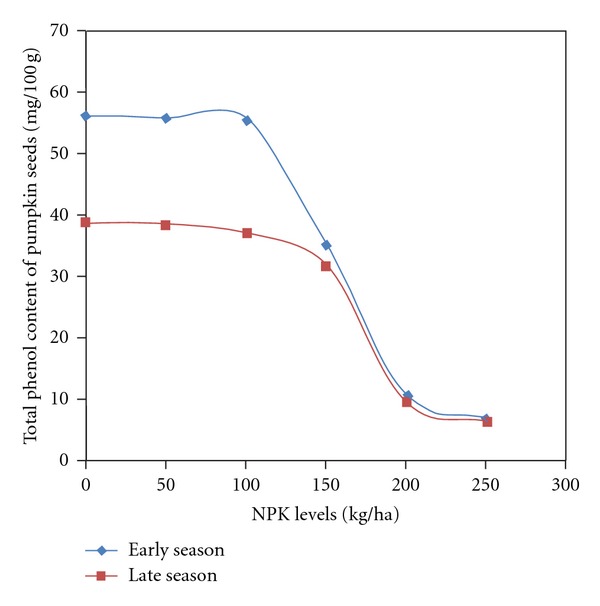
Total phenol and proanthocyanidin content of pumpkin seeds as affected by season by NPK fertilizer.

**Figure 6:**
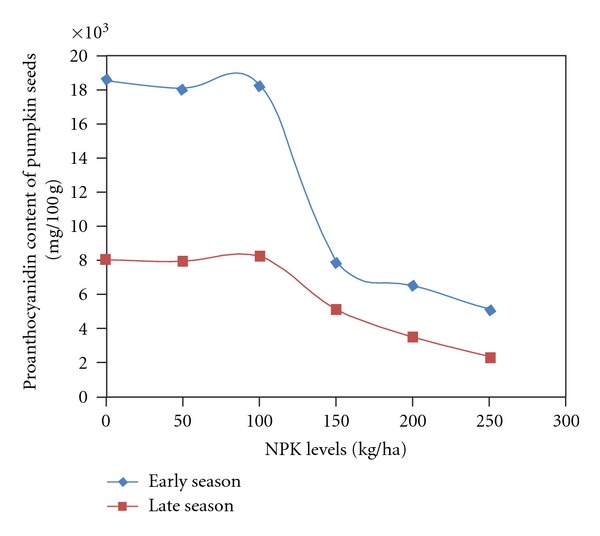
Proanthocyanidin content of pumpkin seeds as affected by season by NPK fertilizer.

Antioxidant activities and its components in pumpkin seeds were significantly influenced by season and fertilizer. The interaction of season and fertilizer was significant on antioxidant activities, phenol, and proanthocyanidin (Table 4). Season had a greater influence on antioxidant activities of pumpkin seeds; however, flavonoid content was not significantly affected by season. Seasonal variation revealed higher values during the early season in all the determined profiles when compared to the late season. The values in flavonoid were similar in both seasons (Table 5). Fertilizer influenced showed consistent decrease in values of antioxidant activities and its components as fertilizer rates increased. The control had significantly higher values than the values from the application of 150 to 250 kg/ha. The addition of 50 and 100 kg gave similar antioxidant activities and component values with the zero fertilization at 0.05 probability level (Table 6). The significant influence of interaction (season × fertilizer) on antioxidant activities, phenol, and proanthocyanidin concentration showed that early season had higher values than late seasons across fertilizer rates. The concentration at both seasons was similar between the control and 50 kg and 100 kg fertilizer. A significant reduction in values was found from 150 to 250 kg NPK fertilizer rate application (Figures 4, 5, and 6).

## 4. Discussion

In this study, application of fertilizer above 100 kg NPK/ha reduced the seed oil yield, fibre, and protein. However, Jariene et al. [3] reported a reduction in seed fat percentage but an increase in seed protein due to fertilizer application, and in the case of crude fibre in seed there was no significant response to fertilizer. Antioxidant properties of *C. pepo* seeds were higher at 0, 50, and 100 kg/ha levels of NPK than all other levels. This corroborated other findings on fruits and vegetables that the use of mineral fertilizer, particularly Nitrogen at high rates, negatively affects the antioxidant properties of fruits and vegetables [15]. The proximate contents and antioxidant properties of pumpkin seeds at control were similar to those of 50 and 100 kg/ha NPK fertilizer rates in this study. This means that at those rates there is a complimentary balance between seed yield and nutrient concentration in the seed. However, there was reduction in antioxidant activities of *C. pepo* seeds under fertilizer rates of 150–250 kg/ha (53–77%) compared with that of 0–100 kg/ha (90%). The result is similar to the reduction in total phenolics and antioxidant activities observed in Mustard leaf due to increased N fertilization. 

 Kader [16] noted that soil type affects antioxidant properties in crops. The crops grown in a sandy soil tend to retain fewer nutrients than those grown in clay soils and hence increase in their antioxidant properties. If the nutrient is low, antioxidant activities is expected to increase. At the University of Illinois comparison was made of flavonoid content in tomatoes under conventional and organic agriculture practices. It was observed that flavonoid levels in tomatoes increased under organic management. Plants with limited N were shown to accumulate more flavonoids than those that are well supplied with inorganic fertilizers. It was concluded that synthetic fertilizers in which N is easily accessible to the plant may reduce the health benefits of tomatoes [17]. This agreed with the observation in this study that overfertilization at 150–250 kg NPK reduces the health benefits of pumpkin seeds. 

 Climatic conditions have a strong influence on the concentration of bioactive compounds [18]. Climatic factors vary with growing sites, seasons, and between years. Temperature, both in terms of total or average temperature and the extremes during the growth period, may influence the chemical composition in plants [18, 19]. In lettuce, light effects increased the concentrations of vitamin C, carotenoids, and flavonoids in the outer leaves than the inner leaves, which receive less light [20–22]. In this study, the pumpkin seeds obtained during the early season had significantly higher proximate contents and antioxidant profile than those of late cropping season. This could be due to excessive rainfall during the late season. High rainfall has been reported to reduce nutrient content and antioxidant composition in vegetable crops [18].

 In conclusion, the cultivation of pumpkin at lower NPK fertilizer rate and under moderate rainfall could enhance the health benefits from pumpkin seeds. World Health Organization had estimated that 2.7 million lives could be saved annually by consuming fruits and vegetables. Hence the consumption and utilization of pumpkin seeds can effectively fill this gap if its potentials are harnessed and improved. 

## Figures and Tables

**Table 1:** Combined analysis of variance showing means squares for protein, fat, ash, crude fibre, and carbohydrate contents of Pumpkin seed as influenced by Season and NPK fertilizer.

Source	DF	Protein (g/100 g)	Fat (g/100 g)	Ash (g/100 g)	Crude fibre (g/100 g)	Carbohydrate (g/100 g)
Season	1	42.40*	3.9204*	0.4959**	0.008438**	85.920**
Rep within season	2	0.16	0.1304	0.00095	0.000254	0.0145
Fertilizer	5	3.24**	1.8218**	0.0494**	0.005278**	11.408**
Season × Fertilizer	5	1.19*	0.0704	0.0111**	0.000258	1.757**
Pooled error	10	0.26	0.0534	0.0005	0.000264	0.210
CV (%)		2.0	0.4	1.5	3.1	3.5

*: significant at 0.05 level of probability.

**: significant at 0.01 level of probability.

**Table 2:** Proximate composition of Pumpkin seeds as affected by season.

Season	Protein (g/100 g)	Fat (g/100 g)	Ash (g/100 g)	Crude fibre (g/100 g)	Carbohydrate (g/100 g)
Early season	27.6	59.0	1.61	0.55	11.3
Late season	24.9	58.2	1.32	0.51	15.1
LSD (0.05)	0.45	0.42	0.008	0.002	0.09

NS = not significant at 5% level of probability.

Values are means of duplicate analyses expressed on dry matter basis.

**Table 3:** Proximate composition of Pumpkin seeds as affected by NPK fertilizer levels.

NPK level (kg ha^−1^)	Protein (g/100 g)	Fat (g/100 g)	Ash (g/100 g)	Crude fibre (g/100 g)	Carbohydrate (g/100 g)
0	27.0^a^	59.2^a^	1.55^a^	0.56^a^	11.7^c^
50	27.0^a^	59.2^a^	1.55^a^	0.56^a^	11.7^c^
100	27.0^a^	59.2^a^	1.58^a^	0.56^a^	11.8^c^
150	25.9^b^	58.1^b^	1.39^b^	0.51^b^	14.2^b^
200	25.6^bc^	58.0^b^	1.37^b^	0.49^c^	14.6^b^
250	24.9^c^	57.8^b^	1.33^c^	0.49^c^	15.4^a^

Means with the same letter in each column are not significantly different at 5% level of probability using Duncan's multiple range test.

**Table 4:** Combined analysis of variance showing means squares for antioxidant activities and its components in Pumpkin seed as influenced by season and NPK fertilizer.

Source	DF	Antioxidant activities (%)	Phenol (mg/100 g)	Flavonoid (mg/100 g)	Anthocyanin (mg/100 g)	Proanthocyanidin (mg/100 g)
Season	1	2227.23*	567473200*	21327079	8618.46*	254.80**
Rep within season	2	24.46	47581426	8545837	522.83	0.205
Fertilizer	5	1071.78**	1439581031**	81034624**	5491.33**	81.54**
Season × Fertilizer	5	117.85**	78169287**	600093	295.96	16.16**
Pooled error	10	18.73	14234829	1634308	296.16	0.35
CV (%)		5.6	11.9	19.2	26.3	6.5

*: significant at 0.05 level of probability.

**: significant at 0.01 level of probability.

**Table 5:** Antioxidant activities and its components in Pumpkin seeds as affected by season.

Season	Antioxidant activities (%)	Phenol (mg/100 g)	Flavonoid (mg/100 g)	Anthocyanin (mg/100 g)	Proanthocyanidin (mg/100 g)
Early season	86.5	36.70	7.59	0.085	0.012
Late season	67.2	26.97	5.70	0.047	0.006
LSD (0.05)	3.9	6.94	NS	0.018	0.0002

NS: not significant at 5% level of probability.

Values are means of duplicate analyses expressed on dry matter basis.

**Table 6:** Antioxidant activities and its components in Pumpkin seeds as affected by NPK fertilizer.

NPK level (kg ha^−1^)	Antioxidant activities (%)	Phenol (mg/100 g)	Flavonoid (mg/100 g)	Anthocyanin (mg/100 g)	Proanthocyanidin (mg/100 g)
0	90.4^a^	47.45^a^	10.95^a^	0.095^a^	0.013^a^
50	89.9^a^	47.12^a^	10.63^a^	0.097^a^	0.013^a^
100	89.9^a^	46.38^a^	10.460	0.098^a^	0.013^a^
150	77.0^b^	33.36^b^	4.5^b^	0.062^b^	0.006^b^
200	61.3^c^	10.02^c^	2.47^bc^	0.024^c^	0.005^c^
250	52.9^d^	6.6^c^	1.32^c^	0.018^c^	0.004^d^

Means with the same letter in each column are not significantly different at 5% level of probability using Duncan's multiple range test.
